# VEGFA Isoforms as Pro-Angiogenic Therapeutics for Cerebrovascular Diseases

**DOI:** 10.3390/biom13040702

**Published:** 2023-04-20

**Authors:** Amanda Louise White, Gregory Jaye Bix

**Affiliations:** 1 Department of Neurosurgery, Clinical Neuroscience Research Center, Tulane University School of Medicine, New Orleans, LA 70112, USA; 2 Tulane Brain Institute, Tulane University, New Orleans, LA 70112, USA; 3 School of Medicine, Tulane University, New Orleans, LA 70112, USA; 4 Department of Neurology, Tulane University School of Medicine, New Orleans, LA 70112, USA; 5 Department of Microbiology and Immunology, Tulane University School of Medicine, New Orleans, LA 70112, USA; 6 School of Public Health and Tropical Medicine, Tulane University, New Orleans, LA 70122, USA

**Keywords:** VEGFA, isoforms, cerebral vasculature, angiogenic therapy

## Abstract

Therapeutic angiogenesis has long been considered a viable treatment for vasculature disruptions, including cerebral vasculature diseases. One widely-discussed treatment method to increase angiogenesis is vascular endothelial growth factor (VEGF) A. In animal models, treatment with VEGFA proved beneficial, resulting in increased angiogenesis, increased neuronal density, and improved outcome. However, VEGFA administration in clinical trials has thus far failed to replicate the promising results seen in animal models. The lack of beneficial effects in humans and the difficulty in medicinal translation may be due in part to administration methods and VEGFA’s ability to increase vascular permeability. One solution to mitigate the side effects of VEGFA may be found in the VEGFA isoforms. VEGFA is able to produce several different isoforms through alternative splicing. Each VEGFA isoform interacts differently with both the cellular components and the VEGF receptors. Because of the different biological effects elicited, VEGFA isoforms may hold promise as a tangible potential therapeutic for cerebrovascular diseases.

## 1. Introduction

The brain is a vital organ, innervating every biological system of the body. While the brain only accounts for 2% of total body weight, it utilizes 25% of all glucose brought into the body, making it one of the highest energy-demanding organs based on its size [1,2]. The cerebrovasculature is a complex network of blood vessels that supplies the brain with nutrients and removes waste from the surrounding environment. It is no wonder that disruption in the cerebrovasculature can lead to several long-term consequences: cognitive impairment, motor dysfunction, and even death. Maintenance of the cerebrovascular network is largely mediated by the Vascular Endothelial Growth Factor (VEGF) family of proteins. One member of the VEGF family, VEGFA, has gained notoriety for use as a pro-angiogenic therapeutic. VEGFA is a growth factor involved in developmental [3], adult, and pathological angiogenesis [4]. Because of its ability as a pro-angiogenic factor, it was theorized that VEGFA would be a viable therapeutic approach for ischemic-related conditions through the growth of new vessels to reestablish blood flow to the ischemic area, thus reducing neuronal cell loss [5,6]. Animal models of stroke have largely shown that the administration of VEGFA has led to increased angiogenesis and infarct volume reduction. On the other hand, clinical studies have thus far shown that VEGFA administration has a minimal therapeutic effect [7].

The lack of beneficial effects in humans and the difficulty in medicinal translation may be due in part to VEGFA being able to increase vascular permeability. Increased vascular permeability can lead to edema or allow in toxins from the surroundings, exacerbating the injury-affected area. While the idea of pro-angiogenic therapy is promising, the undesirable side effects of VEGFA need to be addressed in order for it to continue to move toward clinical use. One way to improve VEGFA pro-angiogenic therapy and lessen the harmful effects of VEGFA is to use the VEGFA isoforms. VEGFA is able to produce several different isoforms through alternative splicing, each of which has its own biological effect and may hold promise as a tangible potential therapeutic for cerebrovascular diseases. Through the use of VEGFA isoforms and by optimizing the route of administration, the clinical use of VEGFA pro-angiogenic therapy can be improved. This review will discuss the differences between VEGFA and its angiogenic isoforms and the promising future of VEGFA pro-angiogenic therapeutics.

## 2. Therapeutic Use of VEGFA

VEGFA is one member of the VEGF family of growth factors, largely known for its ability to promote endothelial proliferation and its role as a potent angiogenic factor [6,8]. VEGFA can be found circulating in the cerebrovasuclature or bound to the heparin-containing molecules of the extracellular matrix (ECM). Binding to heparin-containing molecules makes the ECM an essential reservoir and regulator of the bioavailability of VEGFA [9]. In addition to angiogenesis, VEGFA promotes neuronal survival, vasodilation, and inflammatory cell recruitment [8]. VEGFA elicits its effects by binding to the receptor tyrosine kinases, VEGF receptor (VEGFR) 1 and 2 [10]. VEGFR1 and VEGFR2 dimerize upon ligand binding and can exist as homodimers or heterodimers with one another, contributing to the various pathways and downstream effects that VEGFA is able to promote [11]. Binding to VEGFR2 mediates most of VEGFA’s iconic functions: endothelial cell proliferation and angiogenesis [11], while binding to VEGFR1 promotes immune cell migration into the parenchyma as well as serving a regulatory role, modulating the effects of VEGFR2 [12]. The effects of VEGFA-VEGFR can be further modulated by neuropilin (Nrp) 1 and 2 [13]. Nrp1 and Nrp2 are single pass transmembrane proteins [14] that act as co-receptors to VEGFR1 and 2 [15]. Nrp1 and Nrp2 have short enzymatically inactive intracellular domains [14] that bind secondary proteins to enhance VEGFA signaling [13,14,16]. Activated VEGFR can still function without the neuropilin co-receptors, although the strength of the signal is decreased [13,17]. VEGFA is constitutively expressed at low levels and is quickly upregulated during wound healing in cases of blood vessel damage [18,19], neuronal damage [20], or ischemia [3,8]. 

**Figure 1:**
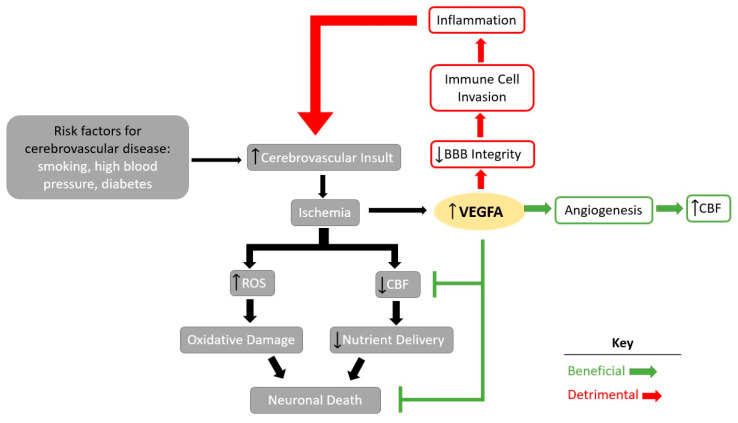
**VEGFA signaling in cerebrovascular diseases.** Vascular endothelial growth factor A (VEGFA)-induced signaling in cerebrovascular disease can lead to both detrimental (red) and beneficial effects (green). BBB: blood–brain barrier. ROS: reactive oxygen species. CBF: cerebral blood flow.

In cerebrovascular disease, VEGFA can have both beneficial and detrimental effects (Figure 1). Risk factors such as smoking, high blood pressure, or diabetes that can lead to cerebrovascular disease [21] and functional impairment of the blood vessels can lead to ischemia of the brain at the site of the injury. Ischemia can then lead to increases in oxidative damage, toxicity, and inflammation, culminating in neuronal death [8]. In response, levels of VEGFA increase to combat ischemic damage by increasing angiogenesis and neuronal protection [4]. However, in cases of severe ischemia, high levels of VEGFA weaken the blood–brain barrier (BBB) integrity, allowing for the invasion of inflammatory cells [8], leading to an increase in inflammation and increased toxicity in the brain, ultimately culminating in neuronal death [22] and further exacerbating the initial injury. Notably, levels of VEGFA also increase with the severity of stroke [23,24], Alzheimer’s Disease (AD) [25], and traumatic brain injury (TBI) [23,25,26,27,28]. This correlation between its upregulation in traumatic vascular events and its role to promote vascular growth, encouraging experimentation with VEGFA as a potential therapeutic. 

With careful consideration for the potential side effects, VEGFA has been administered as a treatment in various animal models for cerebrovascular diseases (Table 1), such as head injuries and stroke. Although, for some publications the distinction between VEGFA or an isoform of VEGFA is not highlighted. Fortunately, VEGFA treatment has been shown to be beneficial to stroke recovery in animal models [29,30,31,32]. The administration of VEGFA resulted in the reduction of lesion size in a mouse model of TBI [26]. In a closed head injury model, VEGFA administration induced angiogenesis, neurogenesis, and improved functional recovery [26]. The administration of VEGFA has also resulted in a decrease in infarct volume, increased angiogenesis [33,34], and improved functional outcomes in rodent models of middle cerebral artery occlusion (MCAO) [33]. A stroke is one of the most important and most common cerebrovascular diseases. Many instances of stroke can lead to long-term functional deficits, including disability and death [35]. Even with the commonly used FDA-approved therapeutic drug, tissue-type plasminogen activator (tPA) [36,37], stroke is still the third leading cause of death and disability worldwide [35]. The high incidence of stroke may be partly due to the fact that tPA must be administered within a few hours after stroke onset in order to be effective [37] and the severely limited easily accessible alternatives. 

Due to the high prevalence of stroke and the short window for effective treatment, it is vital that potential therapeutics to treat cerebrovascular diseases such as stroke continue to be researched. Despite the promising results of in vivo testing, VEGFA treatment in humans, while safe, has so far proved to be not as effective [6,7,45]. In order to overcome the translational hurdle of VEGFA, the ability to promote angiogenesis without disrupting the BBB could be found in the VEGFA isoforms. Although more research on the nature of each of the angiogenic VEGFA isoforms is warranted, current knowledge makes it clear that the isoforms induce different biological effects than their parent molecule [8,43,46]. The varying effects produced by the VEGFA isoforms may be the key in furthering the utility of VEGFA pro-angiogenic therapy for cerebrovascular diseases. 

## 3. VEGFA Isoforms as Therapeutics

**Figure 2:**
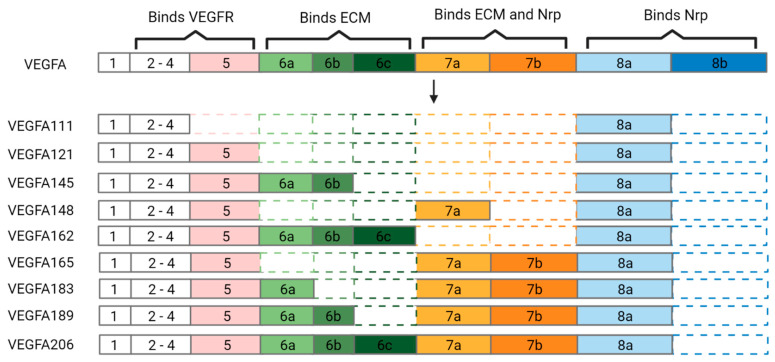
**Comparison of VEGFA to its isoforms.** The eight exons of vascular endothelial growth factor A (VEGFA) have been arranged to highlight occurrences of alternative splicing in each isoform. VEGFR: vascular endothelial growth factor receptor. ECM: extracellular matrix. Nrp: neuropilin. Image created with BioRender.com.

VEGFA contains eight exons; exons 2–8 convey the protein’s binding abilities (Figure 2). Alternative splicing of VEGFA transcripts occurs in regions 5 through 8, generating 9 different pro-angiogenic isoforms with varying interactions. The isoforms are named with the nomenclature ‘VEGFAxxx’, where the ‘x’ corresponds to the number of amino acids each variant contains [47]. In addition to the different mRNA (Figure 2) and protein structures, the VEGFA variants also have unique biological effects and interactions (Table 2). 

### 3.1. VEGFA111

VEGFA111 is a splice variant of VEGFA, containing exons 1–4 and 8a, and is readily secreted upon synthesis [9]. Additionally, 111 promotes angiogenesis, endothelial proliferation [9,47], and is able to bind to VEGFR2 but not to VEGFR1 [50]. The relationship between the neuropilin co-receptors and 111 is currently unknown. However, there is a trend of NRP1 favoring and binding to larger VEGFA isoforms, such as VEGFA189 [16], but whether this means an inability to bind 111 has not yet been shown. Nevertheless, 111 is a unique variant of VEGFA, as it is the only isoform upregulated upon genotoxic stress. Cells exposed to UV-B irradiation and other toxins known to cause DNA damage begin to express 111 more readily than any other VEGFA isoform [50]. In addition, 111 does not increase in response to hypoglycemia and hypoxia, conditions that would both see the upregulation of VEGFA [50]. Overall, 111 is also more resistant to degradation by plasmin when compared to VEFGA165 [50,51]. Taken together, these unique features of 111 imply that it is a long-lasting molecule that plays a role in DNA damage. 

As one of the most unique isoforms of VEGFA, many questions about 111 remain unanswered. Whether 111 is involved in preventing or repairing radiation associated vascular damage, has yet to be determined. Based on the protective role that VEGFA plays in maintaining the cerebrovascular integrity, 111 likely has a role in protecting the vasculature from radiation damage, but more research into the biological role and unique biological effects of 111 should be explored. In the realm of developing therapeutics, if future research shows that 111 is beneficial to recovery from radiation damage, 111 should be explored for use as a therapeutic for damage caused to the cerebrovascular from radiation-induced vasculopathy. 

### 3.2. VEGFA121

VEGFA121 is an isoform of VEGFA-containing exons 1–5 and 8a [9,10]. The isoform 121 promotes angiogenesis, lymphangiogenesis, and increases vascular permeability [47]. 121 is able to bind to both VEGF receptors but has a higher affinity with VEGFR2 than VEGFR1 [10]. Additionally, 121 is able to bind to Nrp2 [15], however it is unclear how 121 interacts with Nrp1. Some studies have reported that 121 is unable to bind to NRP1 [17], while others have reported a low affinity for the co-receptor [15]. In vitro studies show that 121 has a stronger and more rapid angiogenic effect compared to that of VEGFA165 and VEGFA189 [52]. The higher potency towards angiogenesis by 121 may be due to its higher affinity for VEGFR2 and that it is more readily secreted than VEGFA165 and VEGFA 189 [47,52]. VEGFA and some of its isoforms contain heparin-binding domains encoded in exons 6 and 7 that allow them to bind to heparin-containing molecules of the ECM [9,43]. Isoforms of VEGFA that do not contain a portion of exon 6 or 7 are largely thought to be unable to bind to the ECM and are thus freely secreted. Both 121 and VEGFA111 lack the heparin binding domains encoded by exons 6 and 7, making them molecules that are more readily secreted than the other VEGFA isoforms. However, while VEGFA111 is unable to bind to the heparin-containing molecules of the ECM [9,47], there is some disagreement on the binding capabilities of 121. On the other hand, co-immunoprecipitation and western blot analysis of 121 showed weak binding to heparin [49]. Overall, 121′s ability to bind to heparin-containing molecules, despite not having exons 6 and 7, implies that a third heparin binding domain may be encoded in exon 5 as well. A binding assay should be used to determine the binding affinity more accurately for 121 and heparin [10].

Although 121 weakens the BBB, the potential therapeutic benefits may outweigh the detriments at high concentrations. Similar to the mixed and disappointing clinical trials of VEGFA, clinical trials using 121 as a treatment have also shown that its administration is safe [53], while having mixed results in terms of its use as a therapeutic [54,55]. In order to gather more evidence to support the therapeutic use of 121, changes in BBB integrity when exposed to high concentrations of both should be compared to that of VEGFA. Further studies on the biological properties of 121 can help determine its potential as an angiogenic therapeutic.

### 3.3. VEGFA145

VEGFA145 is a splice variant containing exons 1–6b and 8a [9,10]. 145 is only able to bind to VEGFR2 [56]. Since 145 is unable to bind to VEGFR1, and consequently does not have its intrinsic regulation, the use of 145 may have a higher angiogenic potency than the other isoforms. In addition, 145 is able to bind to Nrp2 as well [13]. Therefore, a smaller dosage of 145 may lead to comparable angiogenic effects to the other isoforms at higher concentrations. Notably, 145 is tightly bound to heparin-containing proteoglycans, giving it a tight bond to the ECM [47]. Binding to the ECM functions as a storage for VEGFA and its isoforms. VEGFA or isoforms that are bound to the ECM can be released through the actions of heparinases, an enzyme that cleaves heparin, thus allowing for the regulated release of these molecules and prolonged angiogenic stimulation [10,31,46,50,56]. More studies should be conducted to determine if there is a difference in the strength of the angiogenic effect between matrix bound and free-floating isoforms. Binding to the matrix affects bioavailability and may also affect how long it remains in circulation. Higher doses of free-floating isoforms may be needed, compared to isoforms that bind to the ECM, in order to see a comparable pro-angiogenic effect. 

With its lack of regulation from VEGFR1 and its ability to have the enhancing effects of Npr2, the therapeutic use of 145 may induce a strong angiogenic effect without weakening the BBB or promoting immune cell invasion. However, the strength of the angiogenesis induced by 145 and how it affects BBB integrity is currently unknown. Further studies to characterize the biological effects of this isoform are necessary, as 145 is a strong candidate for a pro-angiogenic therapeutic for cerebrovascular diseases that avoids the detriments seen with VEGFA. 

### 3.4. VEGFA148

VEGFA148 is a splice variant containing exons 1–5, 7a, and 8a [47]. The isoform 148 promotes angiogenesis through binding of VEGFR2 [47], but it is unknown as to whether it can bind VEGFR1. Little is known about 148, but further speculations can be made based on its RNA structure. Because 148 contains a portion of exon 7, it can be inferred that 148 has the ability to bind to the ECM [10] and to the neuropilin receptors. However, the binding strength that 148 has for its receptors has yet to be determined. 

The lack of information currently known about 148 leaves it open for novel findings and therapeutic exploration in cerebrovascular diseases. Additionally, there are still things to be learned about the binding capabilities conveyed by the exons of VEGFA. For 148 and several of the other VEGFA isoforms, their binding capabilities cannot be determined by the exons alone. In other words, it is unclear how alternative splicing within exon 6, 7, and 8 affects the bond strength between VEGFA isoforms and its binding partners. For example, exon 6 and exon 7 both encode the ability for binding to the ECM, but which exon or exon segment is most relevant to a strong bond is unknown.

### 3.5. VEGFA162

VEGFA162 is a pro-angiogenic splice variant containing exons 1–6c and 8a [47], and 162 binds to both VEGFR2 and to the ECM via the heparin-containing molecules contained within [10,47]. Being tightly bound to the ECM, 162, like other VEGFA variants bound to heparin, depends on the activity of heparinase in order to be released into circulation and interact with the VEGF receptors [57]. However, how tightly 162 binds to the ECM and the strength of its angiogenic effect is unknown. Likewise, it is also unclear how 162 interacts with the neuropilin receptors. However, since 162 contains a portion of exon 8, it does have the potential to bind to Nrp1 and or Nrp2. Due to the lack of information currently available about 162, it is hard to predict how it will act as a therapeutic for cerebrovascular diseases. On the other hand, the scarcity of information on 162 leaves it open for novel findings and therapeutic exploration. 

### 3.6. VEGFA165

VEGFA165 is one of the most studied and the most abundantly produced VEGFA isoforms in the body [47]. It has been frequently studied in translational research and in clinical trials, and 165 is a pro-angiogenic splice variant containing exons 1–5, 7a, and 8a [9,10,47,49]. It is known that 165 increases vascular permeability and promotes proliferation of lymphocytes and neurons [47] and is also able to bind to the ECM [58], yet around 50% of 165 can also be found unattached in circulation [3,56]. While 165 is able to bind to both VEGF receptors [47,49] and Neuropilin co-receptors [13,15], there is not a consensus on how strongly it binds to either group. There is evidence that supports 165 having a stronger affinity for VEGFR2 than VEGFR1 [3,56]. There has also been evidence for an equal binding affinity towards both VEGFR1 and 2 [10] and no comparison of binding strength between Nrp1 and Nrp2 [3,56]. The strength of binding of a VEGFA isoform to its receptor is important for determining the potency of its angiogenic effect and to better inform choices around how much to administer as a therapeutic. 

Nonetheless, 165 is one of the few VEGFA isoforms to be used as a treatment for cerebrovascular disease. In vivo studies have demonstrated that administration of 165 leads to improved stroke outcome, enhanced angiogenesis, decreased infarct size, preservation of BBB integrity, and neuroprotection [38,40,52]. In a mouse model using MCAO, treatment with 165 showed increased neurogenesis [59] and angiogenesis [60]. Additionally, 165 treatments were shown to increase levels of perlecan in human brain endothelial cells [61], an effect that has yet to be shown by VEGFA [31]. Perlecan is a heparin-containing proteoglycan present in the ECM [62] that has neuroprotective and proangiogenic properties [60]. Interestingly, combined administration of VEGFA and perlecan improved stroke outcomes in a murine model of stroke [59,60,63]. 165 was able to bind to heparin-containing molecules, and it is reasonable to conclude that it can bind to perlecan as well. Furthermore, perlecan also upregulates VEGFA secretion [63]. Therefore, there may be a positive feedback loop between 165 and perlecan. The interactions between VEGFA and its isoforms with perlecan should be further explored. 

Similar to VEGFA, the promising results from in vitro studies have encouraged the use of 165 in clinical trials as well. While there are not many trials for cerebrovascular diseases, treatment with 165 has promising results in clinical trials for critical limb ischemia (CLI) and heart abnormalities. Using 165 for the treatment of CLI has proved beneficial, resulting in decreased pain, increased vascularization, and increased blood flow [64,65]. Unfortunately, the treatment of heart abnormalities with 165 has had mixed results, with some trials showing beneficial effects and others showing complications. In a clinical trial for coronary angina, patients have benefited from treatment with 165, showing signs of reduced angina and reduced ischemic defects [66]. The therapeutic use of 165 in clinical trials of cardiac artery disease (CAD) has shown safe administration with no beneficial effect. Others show some improvement, increased neovascularization, and reduced angina, with some patients having coronary-related complications in follow-up studies (REF). However, others have reported benefits; administration of 165 reduces angina and improves outcome in patients [67]. One consistent result that a majority of clinical trials have shown, regardless of disease being treated, is that therapeutic use of 165 is safe [68,69]. While improvements still need to be made, therapeutic use of 165 should be encouraged for cerebrovascular disease, as alignments that affect the heart often have an impact on the brain as well. Similarly, treatments that help the heart may also be effective in treating the cerebrovascular. 

### 3.7. VEGFA183

VEGFA183 is an understudied VEGFA isoform, and is a splice variant that promotes angiogenesis and contains exon 1–6a and 7a–8a [47,48]. It also binds to heparin-containing proteoglycans on the ECM and to VEGFR2 [47]. The studies of the interactions between 182, Nrp1, and Nrp2 have not been reported. How tightly it binds to the ECM or either of the neuropilin receptors is unknown. The lack of information currently known about 183 leaves it open for novel findings and therapeutic exploration. 

### 3.8. VEGFA189

VEGFA189 is a splice variant containing exon 1–6b and 8a [10,47,49]. 189 binds tightly to the heparin-containing proteoglycans in the ECM [47,49], VEGFR2 [47], and Nrp1 [16]. The interaction of 189 with VEGFR1 and Nrp2 is currently unknown. 189 is a pro-angiogenic isoform and is thought to play a key role in promoting vascularization during bone repair [47]. Interestingly, reminiscent of VEGFA165, 189 may also have a relationship with perlecan. Lord et al., 2017, demonstrated that the combination of perlecan and 189 was able to enhance wound healing in a rat model [70]. The administration of 189 and perlecan increased angiogenesis and increased expression of ECM components, collagen, and laminin [70]. Perlecan on its own has been shown to be beneficial in enhancing regeneration [71], but does the combination of 189 and perlecan have a synergistic effect? Are they activating separate and distinct pathways or reinforcing the same regenerative associated pathways? Since the administration of perlecan with 189 resulted in therapeutic results, other combination-based therapeutics with VEGFA or their isoforms may also prove beneficial for cerebrovascular diseases. 

### 3.9. VEGFA206

VEGFA206 is a pro-angiogenic splice variant containing exon 1–8a [9,10,47]. Information about the binding properties and cellular effects induced by 206 is sparse. 206 is a pro-angiogenic isoform of VEGFA able to bind to VEGFR2 and can extracellular heparin-containing proteoglycans of the ECM [47,49]. However, whether it binds to VEGFR1 or to the neuropilin receptors is unknown. The lack of information currently known about 206 leaves it open for novel findings and therapeutic exploration. 

## 4. VEGFA Pro-Angiogenic Clinical Trials

VEGFA as a therapeutic is not new to clinical trials and has been examined in various diseases (Table 3). While there have been trials addressing VEGFA treatment for cerebrovascular diseases, a majority of clinical trials using VEGFA pro-angiogenic therapy have been conducted in coronary artery related dysfunctions [67,68,72]. Results from clinical trials using VEGFA therapy to treat cardiac dysfunction range widely from patients having medical complications in follow up examinations [67], no significant benefit [45], and even improvements in disease outcome [64]. The varying results from these clinical trials have, in part, likely encouraged the recent focus on assessing the reliability and safety of VEGFA administration. Luckily, many recent trials focusing on the utility of VEGFA pro-angiogenic therapeutics, have shown that administration of VEGFA in humans is safe [55,68]. Now, with proven safe administration, focus can move back to improving the therapeutic aspect of pro-angiogenic therapy. 

From examining the few promising VEGFA Pro-angiogenic clinical trials that reported improvements in patient outcome, one commonality among them is the use of direct administration. One clinical trial treating coronary angina administered VEGFA via intramyocardial injection which resulted in a reduction in angina and ischemic defects [66]. Intramyocardial injection of VEGFA also resulted in increased rates of cardiac perfusion and oxygen consumption in a clinical trial for ischemic cardiomyopathy [48]. Another clinical trial for CLI used intramuscular injection of VEGFA and achieved improved patient outcome [65]. These successful VEGFA pro-angiogenic clinical trials that resulted in improvements in patient health all used direct administration methods close to the site of injury. On the other hand, one common feature among pro-angiogenic VEGFA clinical trials with more disappointing outcomes used indirect administration methods. One clinical trial of CAD that administered VEGFA through a catheter found patients that developed coronary related complications [67]. Another clinical trial for CAD that had no beneficial effect, administered VEGFA through an intravenous injection [55]. These unsuccessful VEGFA pro-angiogenic clinical trials used indirect administration methods, injecting VEGFA in places other than the disease or injury afflicted site. Furthermore, Stewart et al., 2006 highlights that their study to treat CAD, was improved by changing administration methods to direct intramyocardial delivery instead of intravenous injection [54]. While Stewart et al., 2006 clinical trial did not report the glowing results expected of a successful clinical trial, it can be further improved by not only changing the route of administration, but the timing and dosage as well.

## 5. Improving VEGFA Administration: Timing, Route, and Dosage

Clinical trials have yet to replicate the beneficial effects of VEGFA treatment to the extent seen in animal models, but several clinical trials have shown that administration of VEGFA, VEGFA121, and VEGFA165 are safe [45,53,65]. VEGFA pro-angiogenic therapy can be further improved for treatment of cerebrovascular diseases by increasing dosage, targeted administration to the afflicted area, and consideration of the timing of administration. Comparisons of both in vivo and clinical trials show that changes in the timing, dosage, and route of VEGFA administration can have differences in therapeutic effects and a worsening of symptoms. 

Considerations of timing of administration can improve therapeutic outcome by addressing the pathological changes that occur over time. Similar to methods used in in vivo stroke therapeutics, one way to improve beneficial effects, is to treat both the acute and late phases. Intravenous administration of VEGFA during the acute phase of stroke, 1–24 h [6], leads to an increase in infarct size and BBB permeability [34,52]. On the other hand, if VEGFA is administered intravenously in the late phase of stroke, 24 h after stroke onset [6], infarct size decreases [34]. For in vivo models of stroke, the difference between worsening effects and improved outcome can be influenced by when therapeutic intervention occurred. In a study by Manoonkikiwongsa et al., 2006 comparing the differences of stroke outcome between a high and low dose of VEGFA, discovered that timing played a key role. A low dose (2 µg) of VEGFA165 administered intravenously during the early phase of stroke minimized the damage caused by MCAO significantly better than a high (60 µg) dosage [34]. Low dosages of VEGFA, while not inducing angiogenesis, remains neuroprotective [6,34]. Further studies on the biological changes in cerebrovascular diseases that occur throughout disease progression should be conducted in order to determine the optimal windows of treatment or how each individual phase can be treated separately. 

Route of VEGFA administration also makes a difference in therapeutic effect. In in vivo models of stroke, topical application or intracerebroventricular application prevents neural damage as well as BBB permeability [6]. Mice that have undergone an MCAO procedure showed better improvement when injected with VEGFA165 in an intracerebroventricular application as opposed to intravenous injection [38]. Intracerebroventricular injected mice showed less damage to the BBB and had a smaller infarct region compared to their intravenously injected counterparts [38]. Direct application of VEGFA to the brain by surgically lifting a part of the skull to treat stroke has also been effective [40,41]. In clinical trials of CAD, changing administration methods from intravenous to intramyocardial has led to an overall improvement in results. 

For cerebrovascular disease, intracerebroventricular administration may lead to a diminished infract, but the surgery needed for such a direct injection method can also be an invasive process [76]. Alternative methods for close administration to target the cerebral vasculature that also minimizes risk can be found in methods like intranasal administration [76] and systemic administration. Intranasal application allows drugs to reach the brain by direct transport from the olfactory region and has been successfully shown using VEGFA [76]. Other targeted administration methods, such as intravitreal injection and spinal fluid injection, should be tested to determine if it enhances VEGFA therapeutic outcome. 

Considerations of dosage is important for all therapeutics. Optimal dosage for VEGFA is a task that is still being studied and is especially important when using VEGFA. Depending on the dosage, VEGFA can either be neuroprotective and stabilize the BBB or it can weaken the BBB and increase the chances of neuronal death [34]. More information on the VEGFA isoforms needs to be garnered before any similar distinctions on dosages can be made. Due to the different interactions with the ECM in addition to the differences to which angiogenesis in induced, it is reasonable to assume that administration of different amounts of each isoform will be required to reach a therapeutic effect. 

## 6. Considerations for the Future

Therapeutic interest in VEGFA derived from its upregulation during ischemic events and its role as a prominent pro-angiogenic factor. VEGFA has shown beneficial effects in animal models of cerebrovascular diseases like stroke and TBI, but similar beneficial effects have yet to be observed in humans. One way to overcome the translational barriers of VEGFA pro-angiogenic therapy and improve patient outcome is to use VEGFA isoforms. Each VEGFA isoform interacts differently with the VEGF and neuropilin receptors. Differences in receptor interaction gives each VEGFA isoform unique and varying biological effects that can be manipulated and optimized for treatment of cerebrovascular diseases. Isoforms like VEGFA145, that do not bind to VEGFR1, may induce a stronger angiogenic response than the other isoforms, possibly even of VEGFA itself. On the other hand, VEGFA111 might be a better isoform to use if cerebral vasculature disruption is caused by UV damage. Or combination therapy using VEGFA189 and perlecan may result in a strong desirable neuroprotective effect. If VEGFA administration results in increased BBB permeability, then replacing it with VEGFA183 may provide comparable benefits from angiogenesis while preserving BBB integrity. The differences between the VEGFA isoforms may indicate distinct biological roles for them as well. More studies comparing the different biological effects of each isoform to one another and to VEGFA can help clarify their strengths and weakness. One isoform of contention that is not discussed in this review is VEGFAx. Sadeepa Eswarppa et al., 2014 identified the isoform VEGFAx in 2014 [77]. VEGFAx is produced from VEGFA through programed translational read through, a process that extends the proteome pass the canonical stop codon [77]. Currently, there are two studies showing that VEGFAx has anti-angiogenic [77] or pro-angiogenic properties [78]. In order to understand the nature of this new VEGFA isoform, as well as the others, further research into its biological effects is needed.

Further exploration into using VEGFA isoforms is one way to improve VEGFA pro-angiogenic therapy to treat cerebrovascular diseases. Another avenue to be examined to improve VEGFA therapeutics is by using methods of direct administration. Multiple studies in animal models and in clinical trials have demonstrated that direct administration in VEGFA pro-angiogenic therapy improves therapeutic effect. In addition to how it is administered, when administration takes place is also important. Similar to tPA that has a short window of effect during the early phase of stroke before it becomes detrimental, VEGFA isoforms may have similar constraints or they may have multiple periods where there therapeutic use can be maximized. Perhaps, the co-administration of VEGFA, will even extend the therapeutic window of tPA. Taken together, use of a VEGFA isoform that has minimal effect on BBB integrity, with a strong angiogenic and neuroprotective effect administered close to the site of injury will produce a high therapeutic effect.

VEGFA therapy is ideal for treating cerebrovascular diseases, where the main issue is vascular dysfunction or injury. If VEGFA induced angiogenesis can be used to grow new functioning blood vessels to replace the dysfunctional ones, patients suffering from cerebrovascular disease can be treated and patient outcome improved. Moreover, VEGFA therapy may be beneficial in slowing the progression of Alzheimer’s Disease (AD) and Parkinson’s Disease (PD), where vascular weakness further exacerbates the disease through further neuronal death [8]. AD and PD are neuronal degenerative diseases marked by the loss of neurons and amyloid buildup. The neuroprotective effects of VEGFA may be able to slow neuronal loss. Treatment with VEGFA and a glial cell line-derived neurotrophic factor (GDNF) in 6-OHDA mouse model of AD increased neuronal density [43]. VEGFA polymorphism has also shown promise as a protective marker for Alzheimer’s disease [46]. Two VEGFA single nucleotide polymorphisms (rs7043199*rs6993770 and rs2375981*rs34528081), both of which have been associated with increased VEGFA secretion, are strong protective factors against AD [46]. While VEGFA isoforms hold a promising future in therapeutics, more research regarding their pharmacokinetics and their biological effects is needed. If the right combination of administrative tactics for VEGFA and its isoforms is found, then VEGFA pro-angiogenic therapy could be beneficial for any ailment involving disruption to the cerebral vasculature.

## Figures and Tables

**Table 1:** **Varying effects of VEGFA pro-angiogenic therapy in animal models.** Experimental results of treatment with VEGFA or its isoforms in different disease and animal models lead to an array of different findings. MCAO: Middle Cerebral Artery Occlusion. tMCAO: transient MCAO. BBB: blood–brain barrier. AAV: adeno-associated virus. CHI: closed head injury. mTBI: Mild Traumatic Brain Injury. GDNF: growth-derived neurotropic factor. BDNF: bone-derived neurotropic factor.

Drug.	Animal Model	Disease Model	Dosage	Timing	Route	Result	Reference
VEGFA164	mice	MCAO	8 ng	1 h post	intracerebroventricular	Reduced infarct, increase BBB integrity	[38]
VEGFA164	mice	MCAO	8 ng	3 h post	intracerebroventricular	Reduced infarct, reduced apoptosis, increase BBB integrity	[38]
VEGFA164	Mice	MCAO	15 µg/kg	1 h post	intravenous	Increase BBB permeability	[38]
VEGFA	Mice	CHI	0.84 µg	Same time	Intravenous	Decrease lesion volume, improved neurological severity score	[26]
VEGFA	Rats	spinal cord injury	500 ng	same time	Intraspinal injection	Increased neuronal stem cell proliferation	[20]
VEGFA165	Rats	tMCAO	---	8 weeks before	AAV-plasmid injection	Intracranial hypertension, secondary ischemic injury	[39]
VEGFA	Mice	MCAO	---	28 days before	Conditional activation of transgene	infarct reduction	[29]
VEGFA165	Rats	tMCAO	9 ng	Same time	Gelfoam above cerebral cortex	Reduced infarct size	[40]
VEGFA	Rats	photothrombotic	1 × 10^10^ vg	3 days post	microneedle in cerebral cortex	No inflammation, increase angiogenesis, increase neu	[41]
VEGFA165	Rats	6-OHDA	2.5 µg	4 weeks post	Microsphere implantation on cerebral cortex	No change	[42]
VEGFA165 and GDNF	Rats	6-OHDA	2.5 µg: 2.5 µg	4 weeks post	Microsphere implantation on cerebral cortex	Improvement in neuronal density trending	[42]
VEGFA and BDNF	rats	6-OHDA	1.25 µg: 1.25 µg	3 weeks post	Nanosphere on Right striatum	Increased neuronal density	[43]
VEGFA	Rats	6-OHDA	2.5 µg	3 weeks post	Nanosphere on right striatum	No change	[43]
VEGFA	Rats	mTBI	10 µg/mL	Same time	Intracerebroventricular cannulation	Worse cognitive deficits	[44]

**Table 2:** **Comparison of biological effects of VEGFA isoforms to known functions of VEGFA.** Vascular endothelial growth factor A (VEGFA) and its isoforms’ effects on cells, as well as their binding capacities to VEGFR1, VEGFR2, and the ECM. VEGFR: Vascular Endothelial Growth Factor Receptor. ECM: Extracellular Matrix.

VEGFA Forms	VEGFA	111	121	145	148	162	165	183	189	206
Pro-angiogenic	Yes [9,47]	Yes [9,47]	Yes	Yes [9,47]	Yes [9,47]	Yes [9,47]	Yes [9,47]	Yes [9,47]	Yes [9,47]	Yes [9,47]
Binds to VEGFR1	Yes [31]	No [36]	Yes [9]	No [47]	n/a	n/a	Yes [3]	n/a	n/a	n/a
Binds to VEGFR2	Yes [47]	Yes [47]	Yes [47]	Yes [47]	Yes [47]	Yes [47]	Yes [47]	Yes [47]	Yes [47]	Yes [47]
Binds to Nrp1	Yes [13]	n/a	n/a	n/a	n/a	n/a	Yes [13]	n/a	Yes [16]	n/a
Binds to Nrp2	Yes [13]	n/a	Yes [15]	Yes [13]	n/a	n/a	Yes [13]	n/a	n/a	n/a
Binds to ECM	Yes [3]	No [9]	No [3,9]	Yes [9,47]	n/a	Yes [47]	Yes [9,47]	Yes [47]	Yes [9,47]	Yes [9,47]
Freely secreted	Yes [9]	Yes [9]	Yes [3]	No [48]	n/a	n/a	Yes [3]	n/a	No [3]	No [3]
Pro-neovascularization	Yes [9]	n/a	n/a	n/a	n/a	n/a	n/a	n/a	n/a	n/a
Vascular permeability	Increases [9,47]	n/a	Increases [47,49]	n/a	n/a	n/a	Increases [47,49]	n/a	n/a	n/a
neuroprotective	Yes [47]	n/a	n/a	n/a	n/a	n/a	n/a	n/a	n/a	n/a
Pro-neurogenesis	Yes [47]	n/a	n/a	n/a	n/a	n/a	Yes [47]	n/a	n/a	n/a
Immune cell recruitment	Yes [47]	n/a	Yes [47]	n/a	n/a	n/a	Yes [47]	n/a	n/a	n/a

**Table 3:** **Varying effects of VEGFA pro-angiogenic therapy in clinical trials.** Results from clinical trials using pro-angiogenic vascular endothelial growth factor A (VEGFA) therapy to treat patients with different afflictions vary. CLI: Critical Limb ischemia. CAD: coronary artery disease. LVEF: left ventricular ejection fraction. FGF: fibroblast growth factor. G-CSF: granulocyte—colony -stimulating factor. IHD: ischemic heart disease. HGF: hepatocyte growth factor.

Drug	Disease Model	Dosage	Route	Result	Reference
VEGFA165 and HFG	CLI	2.5 mL	Intramuscular injection of a plasmid	VEGF165 increase, decrease pain, increase vascularization,	[64]
VEGF165	CAD	3.8 mg	Catheter insertion of a plasmid	Decreased angina, myocardial perfusion decrease, coronary-related complications diagnosed in follow-up study	[73]
VEGFA	Reduced LVEF	3 mg	Naked mRNA epicardial injection	Safe, no effect	[45]
VEGFA121	CAD	1 mL	Adenoviral epicardial injection	safe	[53]
VEGFA165	Ischemic cardiomyopathy	2 mg	Plasmid intramyocardial injection	Safe, increased cardiac perfusion and oxygen consumption	[69]
VEGF165	CLI	2.4 mg	Plasmid intramuscular injections	Pain reduction, increased blood flow	[65]
VEGF165 and FGF	CAD	0.5 mg	intramyocardial	Safe, no effect	[74]
VEGF165	Coronary angina	125 µg	Plasmid intramyocardial injection	Reduced angina and ischemic defects	[66]
VEGF165	Coronary angina	250 µg	Plasmid intramyocardial injection	Reduced angina and ischemic defects	[66]
VEGFA165 and G-CSF	IHD	60 µg	Plasmid intramyocardial injection	No effect	[75]
VEGFA121	CAD	4 × 10^10^ particle units	Adenoviral intramyocardial injection	Increased neovascularization, coronary-related complications in follow up study	[54]
VEGFA121	CAD	---	Intramyocardial injections	Safe, no effect	[55]
VEGFA165	CAD	0.25 mg	Plasmid intramyocardial injection	Reduction in angina, safe	[67]
VEGFA165	IHD	0.5 mg	Plasmid intramyocardial injection	Trend towards improvement	[72]
VEGFA165 and HGF	CLI	---	Plasmid intramuscular injection	Safe, no effect	[64]
VEGFA165	Coronary angina		Intravenous injection	No effect	[7]

## Data Availability

Not applicable.
